# Inferior vena cava distensibility during pressure support ventilation: a prospective study evaluating interchangeability of subcostal and trans‑hepatic views, with both M‑mode and automatic border tracing

**DOI:** 10.1007/s10877-024-01177-8

**Published:** 2024-05-31

**Authors:** Mateusz Zawadka, Cristina Santonocito, Veronica Dezio, Paolo Amelio, Simone Messina, Luigi Cardia, Federico Franchi, Antonio Messina, Chiara Robba, Alberto Noto, Filippo Sanfilippo

**Affiliations:** 1 2nd Department of Anaesthesiology and Intensive Care, Medical University of Warsaw, Warsaw, Poland; 2 Department of Anaesthesia and Intensive Care, A.O.U. Policlinico-San Marco, Via S. Sofia N 78, 95123 Catania, Italy; 3 School of Anaesthesia and Intensive Care, University “Magna Graecia”, Catanzaro, Italy; 4 Department of Human Pathology of Adult and Childhood “Gaetano Barresi”, University of Messina, Messina, Italy; 5 Cardiothoracic and Vascular Anesthesia and Intensive Care Unit, Department of Medical Science, Surgery and Neurosciences, University Hospital of Siena, 53100 Siena, Italy; 6 Humanitas Clinical and Research Center - IRCCS, Milan, Italy; 7 Department of Biomedical Sciences, Humanitas University, Pieve Emanuele, MI Italy; 8 Department of Surgical Science and Diagnostic Integrated, University of Genoa, Genoa, Italy; 9 IRCCS Ospedale Policlinico San Martino, Genoa, Italy; 10 Division of Anesthesia and Intensive Care, Policlinico “G. Martino”, Messina, Italy; 11 Department of Surgery and Medical-Surgical Specialties, University of Catania, Catania, Italy

**Keywords:** Artificial intelligence, Vexus, Correlation, Echocardiography, Fluid responsiveness

## Abstract

The Inferior Vena Cava (IVC) is commonly utilized to evaluate fluid status in the Intensive Care Unit (ICU),with more recent emphasis on the study of venous congestion. It is predominantly measured via subcostal approach (SC) or trans-hepatic (TH) views, and automated border tracking (ABT) software has been introduced to facilitate its assessment. Prospective observational study on patients ventilated in pressure support ventilation (PSV) with 2 × 2 factorial design. Primary outcome was to evaluate interchangeability of measurements of the IVC and the distensibility index (DI) obtained using both M-mode and ABT, across both SC and TH. Statistical analyses comprised Bland–Altman assessments for mean bias, limits of agreement (LoA), and the Spearman correlation coefficients. IVC visualization was 100% successful via SC, while TH view was unattainable in 17.4% of cases. As compared to the M-mode, the IVC-DI obtained through ABT approach showed divergences in both SC (mean bias 5.9%, LoA -18.4% to 30.2%, ICC = 0.52) and TH window (mean bias 6.2%, LoA -8.0% to 20.4%, ICC = 0.67). When comparing the IVC-DI measures obtained in the two anatomical sites, accuracy improved with a mean bias of 1.9% (M-mode) and 1.1% (ABT), but LoA remained wide (M-mode: -13.7% to 17.5%; AI: -19.6% to 21.9%). Correlation was generally suboptimal (r = 0.43 to 0.60). In PSV ventilated patients, we found that IVC-DI calculated with M-mode is not interchangeable with ABT measurements. Moreover, the IVC-DI gathered from SC or TH view produces not comparable results, mainly in terms of precision.

**Supplementary Information:**

The online version contains supplementary material available at 10.1007/s10877-024-01177-8.

## Introduction

Assessment of the inferior vena cava (IVC) is a central part of many ultrasound-guided fluid management protocols. It has been mainly used for the assessment of fluid responsiveness (FR). However, after an initial enthusiasm due to relatively fast learning curve, it appeared that its predictive value in critically ill patients is suboptimal [1–3]. More recently, the study of the IVC has been included in algorithms attempting the quantification of venous congestion. The main goal of volume expansion with intravenous fluid in critically ill patients is to increase their cardiac output and ultimately optimize perfusion and the delivery of oxygen to the organs, without causing additional harm related to the fluid overload. Both Hypovolemia might be responsible for a reduced preload, stroke volume and ultimately decreases organ perfusion [4–6]; however, an excess of intravenous fluid administration may promote venous congestion—interstitial edema and congestion at pulmonary and/or systemic level [7–9], hence, both conditions are associated with increased mortality in the intensive care unit (ICU) [10]. Recent evidence highlights that a non-negligible proportion of fluid-responder patients may be not fluid tolerant, due to signs of venous congestion [11]. The IVC assessment, whether it is performed for the evaluation of FR and/or venous congestion, is feasible in most clinical settings. However, there are several limitations when using the IVC indexes of FR, namely the IVC collapsibility index (CI) in case of spontaneous breathing with negative pressure, and/or the IVC distensibility index (DI) under positive pressure ventilation. The subcostal approach (SC, also known as sagittal view) is the traditional method to visualize the IVC, though it may not be practical in several scenarios, such as in morbidly obese patients, individuals after laparotomy surgical incision or with in situ chest drains, or those with dilated bowel. Alternatively, the trans-hepatic method (TH, also known as coronal or right lateral) approach has been proposed, and it offers a latero-lateral IVC visualization. There is conflicting evidence on the interchangeability of use of these two views [12–15], and uncertainties have been confirmed by a systematic review [16]. Hence, the TH view remains not validated and the cut-offs used with data gathered from IVC in SC view cannot be adopted.

Meaningfully, the use of artificial intelligence is rapidly expanding across various medical contexts, particularly in echocardiography. Artificial intelligence has been incorporated to assess not only left and right ventricular function [17–20], but also in the assessment of valvular pathology [21] and congenital heart anomalies [22]. Preliminary data supports also the implementation of machine learning programs for the IVC assessment with automated border tracking (ABT) [23].

We previously studied healthy volunteers and ICU patients supported with pressure-controlled ventilation [14, 15]. Overall, comparison between ABT and M-mode (both in SC and TH windows) showed good accuracy but low precision in both studies. When comparing SC view with the TH window, we also found good accuracy and low precision in mechanically ventilated patients [14], but accuracy in healthy volunteers was not acceptable [15], suggesting greater challenges in the use of IVC in spontaneous breathing patients, as the breathing pattern itself may greatly influence results [24].

We conducted a pre-planned third study with prospective observational design on intubated ICU patients with assisted breathing on pressure-support ventilation (PSV) mode, aiming at comparison of differences in IVC measurements taken in M-mode or ABT approach, as well as those obtained from different anatomical sites (SC and TH). We hypothesized that SC and TH views do not provide interchangeable results; we also postulated that ABT may be a valid clinical asset with fair reproduction of M-mode imaging.

## Materials and methods

This is a prospective observational single center study. Our research received approval from the local Ethical Committee (Reference protocol: 53/2022/PO) before patient enrolment. We evaluated differences between the dimensions of the IVC in two methods of visualization (SC and TH windows) in critically ill adult patients supported by PSV mode admitted to the *Azienda Ospedaliera Universitaria Policlinico G Rodolico – San Marco”, Catania (Italy)*.

### Participants

Patients were included if they were intubated and assisted in PSV with inspiratory support set at 6–8 ml/kg of predicted body weight and a positive end-expiratory pressure (PEEP) ≤ 8 cmH_2_O, and under stabilized and steady hemodynamic conditions (defined arbitrarily as no changes in vasopressor dose in the previous four hours). From neurological perspectives, patients with fluctuating sedation level were not included. Although we did not formally record the sedation score, most of them were still sedated at the time of imaging. We followed as much as possible the PRICES guidelines [25, 26].

### Study procedure

At the time of the ultrasound examination, all patients were in a semi-recumbent position (30–40°). One operator with advanced echocardiography certification (FS) performed all scans acquiring four types of visualizations. In particular, the SC and TH windows were acquired at the respective anatomical sites, with two approaches for calculation of IVC diameters: standard M-mode or with ABT. Images were all acquired using the *General Electric (GE) Venue Go R2 device* holding a tracking functionality called *“auto IVC”,* automating the assessment of the vessel’s diameter and providing the IVC-DI and IVC-CI depending on patterns of ventilation (selection made by the operator). The sequence of imaging was not randomized, as M-mode image was always recorded before activating the ABT function, to avoid bias and influence from the values appearing on the screen when ABT mode is active. Despite the IVC variation is influenced by depth of inspiration [24] and by diaphragmatic excursion [27], images were taken within seconds so that it is reasonable to assume that patient’s contribution in the pattern of breathing was constant. The operator acquired images as close as possible to the right atrium and within 4 cm of the cavo-atrial intersection. Attempts were made by the operator to minimize errors due to the cranio-caudal displacements of the vessel during the respiratory cycle, aligning the target sector of the IVC in the center of the screen so the M-mode angle would be as close to 90° as possible.

### Off-line calculation procedure

After the sonographic assessment, the images obtained were downloaded from the ultrasound machine and saved in a password-protected file. After the entire cohort of patients was recruited, in a single session the operator performed an off-line calculation of the IVC diameters and DI obtained with M-mode; when calculating the parameters from M-mode imaging, he was blinded from the ABT results. Conversely, the values of the IVC derived with ABT approach were directly obtained at the bedspace using the software tracking IVC boundaries, with each record lasting six seconds, with several images collected and stored. After the exam the operator performed a check for artifacts and errors before calculating the M-mode IVC diameters and DI, to avoid any bias.

### Study groups and outcomes

We used a 2 × 2 methodological design, allowing to compare the interchangeability between: a) modalities of calculation (in M-mode or with ABT) from images obtained with a single sonographic window (SC or TH), or b) values calculated with the same modality (M-mode or ABT) from images obtained in different sonographic windows (SC vs TH).

Data obtained were divided in 4 different groups: a) SC on ABT; b) SC on M-mode; c) TH on ABT; and d) TH on M-mode. We planned as primary outcome to use the IVC-DI as the intrathoracic pressures of patients in PSV are positive throughout the respiratory cycle. Secondary outcomes evaluated were the interchangeability of IVC min and max diameters with measurements performed with M-Mode and ABT strategy.

### Statistical analysis

Previous investigations evaluating the interchangeability of the SC/TH views showed highly variable results (Pearson coefficient, comprised between values of r from 0.14 [28] to 0.86 [29]). A systematic literature review highlighted that the agreement between the two views was poor to moderate [16]. Therefore, we calculated the necessary sample with a statistical power set of 80% and an alpha level at 0.05, estimating the correlation at r = 0.60. Estimated sample size was 19. Accounting for patients with no SC or no TH windows, we decided to increase the sample size to 23. Concerning the interchangeability between M-Mode and ABT calculations, literature on this correlation is rather limited. As a result, we did not determine a sample size specifically for that aspect of the study; instead, we relied on the recruited sample to perform comparisons between SC and TH windows. We computed the mean bias and limits of agreement [LoA] for IVC diameters and IVC-DI using the Bland–Altman method. The obtained plots were adjusted to account for the impact of multiple measures when comparing ABT modalities [30]. Our analyses are described with mean bias and LoA coupled with their 95% confidence interval (CI). For the comparison between ABT methods (SC vs TH), we used the Bland Altman with repeated measures calculation, and followed the CI95% calculation as suggested by Zou et al. [30].

As per methodology our previous study on mechanically ventilated patients [14], regarding the accuracy and the precision of the IVC-DI we defined as acceptable or good accuracy a mean bias of 4% and 2% (absolute values), respectively; similarly, for the precision we pre-established a LoA of 16% (acceptable) and 8% (good). To characterize the inter-rater variability among measures obtained using the same modality or approach, we computed the Spearman correlation and the intra-class correlation (ICC) coefficient. Correlation interpretation was conducted based on predefined thresholds [31].

## Results

The baseline characteristics of the enrolled patients are outlined in Table 1. The primary admission diagnosis and individual severity scores are separately presented in the Additional material. In all 23 patients included the IVC was visualized in the SC method, whilst in four patients the TH window was not obtained (17.4%). The mean IVC-DIs were 16.3 ± 9.5% (SC) and 14.0 ± 9.2% (TH). The mean for the overall ABT calculation in SC and TH views were 26.6 ± 29.3% and 27.3 ± 30.3%, respectively.

**Table 1:** Baseline characteristics, ventilatory settings and hemodynamic variables of the population of patients studied whilst ventilated in pressure support ventilation (PSV) mode

Baseline characteristics and measurements (n = 23)		Ventilatory settings and Hemodynamics	
Gender (male, n, %)	18/23 (78%)	PEEP (cmH_2_O)	5[5-6]
Age (years-old)	59[46.5–70.75]	Pressure Support (cmH_2_O)	12 [9–14.5]
Weight (Kg)	75.5 [65–87.25]	Tidal Volume (ml)	460 [430–561.5]
Height (cm)	170 [167–175]	Respiratory Rate (bpm)	14 [11-19]
		Oxygen saturation (%)	99 [98–100]
IVCmin in SC (mm, M-mode)	20.2[18–22.8]	Heart Rate (bpm)	89[70.5–94]
IVCmax in SC (mm, M-mode)	23.7[22–24.8]	Sinus rhythm (n, %)	22/23 (95.7%)
IVC-DI in SC (%, M-mode)	12.9[9.6–20]	Systolic Arterial Pressure (mmHg)	126 [107.5–132]
IVCmin in TH (mm, M-mode)	19.2[16.9–23.3]	Mean Arterial Pressure (mmHg)	78 [73–85]
IVCmax in TH (mm, M-mode)	21.1[20–25.1]	Diastolic Arterial Pressure (mmHg)	57 [52.5–62]
IVC-DI in TH (%, M-mode)	10 [7–17.8]	Pulse Pressure Variation (%)	8 [7–9.25]
SOFA score	10 [9.5–14]	Norepinephrine (mcg/kg/min)	0 [0–0.09]
Mortality (n, %)	10/23 (43%)	Norepinephrine (n, %)	7/23 (30%)

*AI* artificial intelligence, *DI* distensibility index, *IVC* inferior vena cava, *PEEP* positive end-expiratory pressure, *SC* sub-Costal imaging, *SOFA* sequential organ failure assessment score, *TH* trans-hepatic imaging

The outcomes of the Bland–Altman analysis are detailed in Table 2, including mean bias, LoA (with lower and upper bound) and the Spearman rho and the ICC to convey the degree of resemblance between measurements. All values are indicated with their 95%CI. Overall, the lowest mean bias for IVC-DI (primary outcome) was found comparing the ABT images in the two anatomical regions (SC and TH), but with large LoA, as for all the other evaluations. The comparison of IVC-DI values obtained in ABT and M-mode yielded large discrepancies, as shown by a mean bias around 6% for both the SC and the TH windows of measurement, and again wide LoA.

**Table 2:** Summary of comparisons between measurement of the inferior vena cava (IVC) in adult patients mechanically ventilated in pressure support mode. In case of the IVC size analysis in M-mode (M), we analyzed a single measure which was the most reliable measure as decided by the experienced operator performing the calculations. In case of the analysis with automated border tracking (ABT), repeated measures were taken and saved in the database. Results of IVC distensibility index, minimum and maximum diameters (IVC-DI, IVC min and IVC max, respectively) are provided in term of mean Bias and limits of agreement (LoA) with their relative 95% confidence interval (CI), where appropriate. We also provide correlation with rho and intraclass coefficient (ICC) to describe how strong the measurements resemble each other

Comparison	Variable	Mean Bias 95%CI	Upper LoA 95% CI	Lower LoA 95% CI	rho 95%CI	ICC 95%CI
SC ABT vs M	***IVC Min***	*-2.9;* *-3.9 to -1.9*	*1.3;* *-0.4 to 3.1*	*-7.1;* *-8.9 to -5.4*	*0.73;* *0.44 to 0.88*	*0.86;* *-0.02 to 0.96*
	***IVC Max***	*-1.9;* *-2.8 to -0.9*	*2.0;* *0.4 to 3.7*	*-5.8;* *-7.4 to -4.2*	*0.90;* *0.78 to 0.96*	*0.90;* *0.45 to 0.97*
	***IVC DI (%)***	***5.9;*** ***0.2 to 11.5***	***30.2;*** ***20.4 to 40.0***	***-18.4;*** ***-28.4 to -8.6***	***0.43;*** ***0.01 to 0.73***	***0.52;*** ***-0.18 to 0.82***
TH ABT vs M	***IVC Min***	*-3.5;* *-4.7 to -2.4*	*0.7;* *-1.3 to 2.8*	*-7.8;* *-9.9 to -5.8*	*0.84;* *0.60 to 0.94*	*0.83;* *-0.16 to 0.96*
	***IVC Max***	*-2.9;* *-3.7 to -2.0*	*0.2;* *-1.3 to 1.6*	*-5.9;* *-7.4 to -4.5*	*0.84;* *0.59 to 0.94*	*0.87;* *-0.13 to 0.97*
	***IVC DI (%)***	***6.2;*** ***2.3 to 10.0***	***20.4;*** ***13.6 to 27.1***	***-8.0;*** ***-14.7 to -1.3***	***0.60;*** ***0.15 to 0.84***	***0.67;*** ***-0.01 to 0.89***
M SC vs TH	***IVC Min***	*0.4;* *-1.9 to 2.8*	*10.1;* *6.0 to 14.3*	*-9.2;* *-13.4 to -5.1*	*0.42;* *0.01 to 0.75*	*0.68;* *0.15 to 0.87*
	***IVC Max***	*1.0;* *-1.3 to 3.3*	*10.3;* *6.3 to 14.2*	*-8.2;* *-12.2 to -4.3*	*0.32;* *-0.11 to 0.70*	*0.64;* *0.09 to 0.86*
	***IVC DI (%)***	***1.9;*** ***-1.9 to 5.7***	***17.5;*** ***10.8 to 24.2***	***-13.7;*** ***-20.3 to -7.0***	***0.42;*** ***-0.03 to 0.73***	***0.50;*** ***-0.25 to 0.80***
ABT SC vs TH	***IVC Min***	*1.3;*	*9.2;* *7.0 to 12.7*	*-6.5;* *-10.1 to -4.3*	*0.68;* *0.58 to 0.76*	*0.84;* *0.55 to 0.94*
	***IVC Max***	*2.0;*	*9.8;* *7.5 to 13.5*	*-5.9;* *-9.5 to -3.5*	*0.47;* *0.34 to 0.59*	*0.58;* *-0.06 to 0.84*
	***IVC DI (%)***	***1.1;***	***21.9;*** ***17.7 to 28.3***	***-19.6;*** ***-26.0 to -15.4***	***0.59;*** ***0.48 to 0.69***	***0.83;*** ***0.76 to 0.87***

In case of the IVC size analysis in M-mode (M), we analyzed a single measure, whilst repeated measures were obtained with the artificial Automatic Boarder Tracking (ABT). Results of IVC distensibility index (IVC-DI), and on minimum and maximum diameters are reported in terms of mean bias and limits of agreement (LoA) with their relative 95% confidence interval (CI). Spearman’s rho and Intraclass correlation coefficient (ICC) were used to describe the strength of the measurements’ correlation. SC: subcostal; TH: Transhepatic

### Different acquisition modalities

**Fig. 1:**
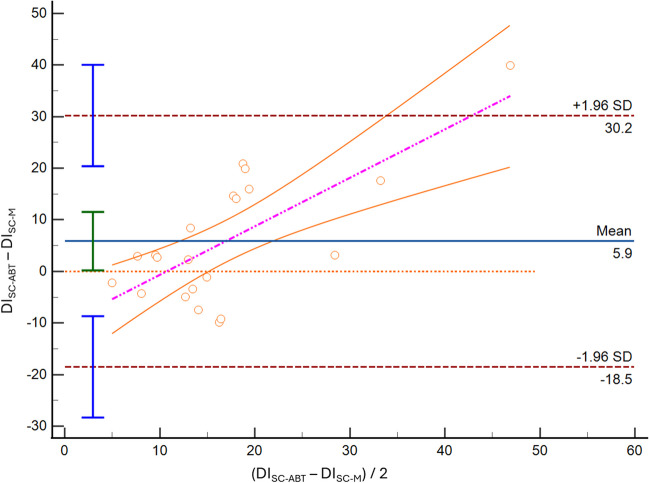
Bland–Altman plot depicting the distensibility index (DI) of the inferior vena cava obtained at the subcostal site, using standard M-mode (SC-M) and artificial intelligence (SC-ABT)

**Fig. 2:**
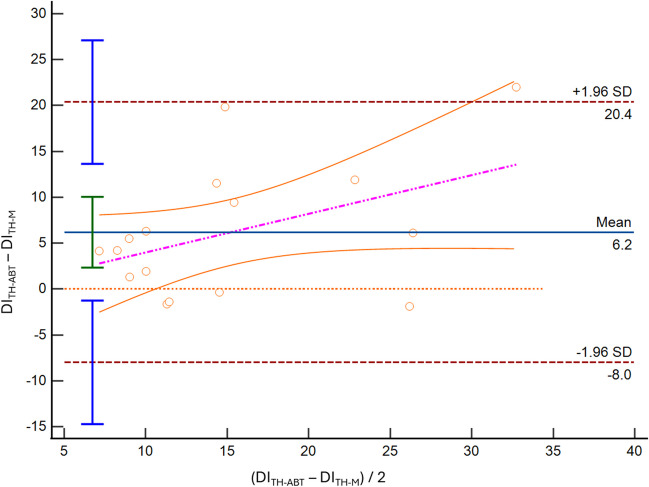
Bland–Altman plot depicting the distensibility index (DI) of the inferior vena cava obtained at the transhepatic site, using standard M-mode (TH-M) and artificial intelligence (TH-ABT)

We found limited accuracy and precision for both SC and TH methods in both modalities of acquisition. Particularly, for the SC imaging of the IVC we found a DI mean bias of 5.9% [LoA − 18.4; 30.2] (Fig. 1), an IVCmax mean bias -1.9 mm, [LoA − 5.8; 2.0], and an IVCmin mean bias -2.9 mm [LoA − 7.1; 1.3]. Comparing M-mode and ABT in the TH, we found an IVC-DI bias 6.2% [LoA − 8.0; 20.4] (Fig. 2), an IVCmax mean bias -2.9 mm [LoA − 5.9; 0.2], and an IVCmin mean bias -3.5 mm [LoA − 7.8, 0.7].

The analysis of correlation with Spearman showed poor-to-moderate reliability for the IVC-DI in SC imaging (0.43 [95%CI 0.01, 0.73]), and moderate for the TH visualization (0.60 [95%CI -0.01, 0.89]). Table 2 reports also the ICC coefficients, which were slightly higher.

### Different acquisition sites

**Fig. 3:**
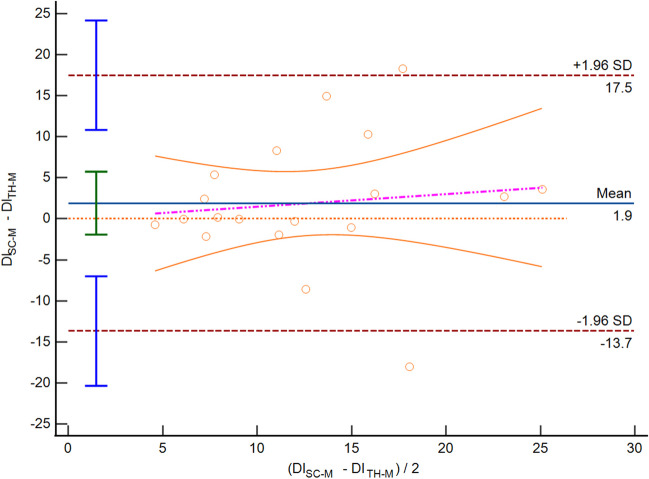
Bland–Altman plot depicting the distensibility index (DI) of the inferior vena cava obtained with standard M-mode, at two different sites: subcostal (SC-M) and transhepatic (TH-M)

**Fig. 4:**
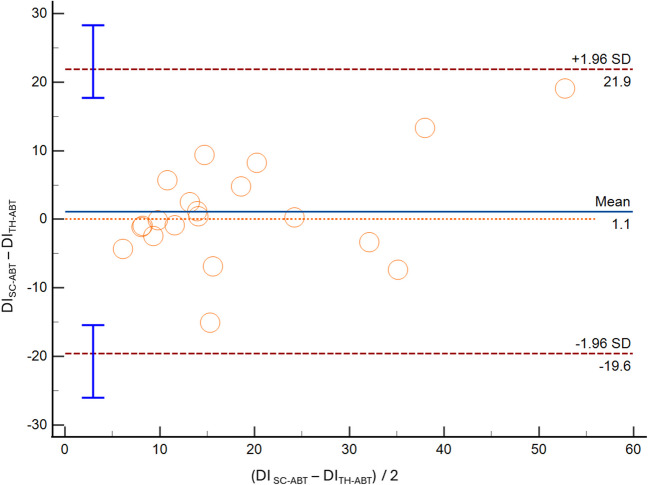
Bland–Altman plot depicting the distensibility index (DI) of the inferior vena cava obtained with artificial intelligence mode, at two different sites: subcostal (SC-ABT) and transhepatic (TH-ABT**)**

In the comparison of IVC assessment between SC and TH methods, we found that in M-mode assessment the IVC-DI showed a 1.9% mean bias [LoA − 13.7; 17.5] (Fig. 3). Likewise, the IVC diameters presented differences between sites of measurement (IVC max: mean bias 1.0 mm [LoA − 8.2; 10.3]; IVC min: mean bias 0.4 mm [LoA − 9.2; 10.1]). With the ABT evaluation, such differences between sites were: IVC-DI mean bias 1.1% [LoA − 19.6; 21.9] (Fig. 4), IVCmax mean bias 2.0 mm [LoA − 5.9; 9.8], and IVCmin mean bias 1.3 mm [LoA − 6.5, 9.2].

Comparing the two anatomical sites, the analysis of correlations with Spearman coefficient between IVC-DIs were moderate for both M-mode (ICC 0.42 [95%CI -0.03, 0.73]) and ABT assessment (0.59 [95%CI 0.48, 0.69]). The ABT assessment showed greater correlation when measured with ICC (0.83 [95%CI 0.76–0.87]).

## Discussion

Our investigation adds new insights to the growing literature on the interchangeability of IVC assessments conducted at two anatomical sites (SC and TH) and also with two different acquisition modalities (M-mode and ABT). The present study follows two previous studies conducted by our group [14, 15], and for ease of interpretation, we summarized the results of these three studies in Table 3. Balancing the findings of these studies it seems apparent that assessment of the IVC from different views or with different measurements approaches yields suboptimal precision, which hampers the interchangeability of results. Moreover, the correlation between calculations is generally poor to moderate.

**Table 3:** A summary of comparisons made between measurements of the inferior vena cava (IVC) in the three studies performed in healthy volunteers, and in patients intubated and ventilated in pressure control or pressure support mode (PCV and PSV, respectively)

Findings from our three studies on IVC assessment with M-mode/ABT, in SC/TH view			SC ABT vs M-mode	TH ABT vs M-mode	M-mode SC vs TH	ABT SC vs TH	Predefined Good	Predefined Acceptable
Intubated and ventilated	**PSV (IVC-DI)**	**Bias**	**5.9**	**6.2**	**1.9**	**1.1**	**2%**	**4%**
		Upper LoA	30.2	20.4	17.5	21.9	***8%***	***16%***
		Lower LoA	-18.4	-8.0	-13.7	-19.6		
	**PCV (IVC-DI)**	**Bias**	** − 3.1**	*** − 2.0***	***0.1***	***2.0***	**2%**	**4%**
		Upper LoA	13.9	*15.4*	*19.3*	*29.7*	***8%***	***16%***
		Lower LoA	− 20.1	* − 19.3*	* − 19.0*	* − 25.7*		
Self-breathing	**Healthy Volunteers (IVC-CI)**	**Bias**	***-0.7***	***3.7***	***13.9***	***7.7***	**4%**	**8%**
		Upper LoA	23.6	*22.3*	*45.8*	34.6	***16%***	***32%***
		Lower LoA	− 24.9	* − 14.9*	* − 18.1*	* − 19.2*		

In all studies, in case of the IVC size analysis in M-mode (M), we analyzed a single measure, whilst repeated measures were obtained with the automated border tracking (ABT) mode. Results of IVC collapsibility and distensibility index (IVC-CI and IVC-DI, respectively), and on minimum and maximum diameters are summarized in terms of mean bias and limits of agreement (LoA) without relative 95% confidence interval. We report also the predefined cut-offs for acceptable or good accuracy (mean bias) or precision (LoA). SC: Subcostal; TH: Transhepatic

The mean bias reported by the present study is similar to the one conducted in patients ventilated in controlled mode for the differences between images acquired at different anatomical sites (SC or TH). This suggests a similar influence of mechanical ventilation on direction of changes in the diameter of IVC. However, slightly greater mean bias (lower accuracy) was seen when comparing these findings acquired at the same anatomical site with different approach (m-mode or ABT). It cannot be excluded that this is due to greater fluctuations in patient’s contribution and influence on breathing, though images were recorded within seconds. The progressive stimulation produced by the pressure of the ultrasound probe during the course of the exam may have produced ups and downs in the level of consciousness and breathing contribution of the patient, with variable interaction with the ventilator.

In general, it is likely an irregular/anisotropic change of IVC diameters during the respiratory cycle, with an elliptical shape occurring in most cases due to the not proportional reduction in antero-posterior (seen in SC) and latero-lateral (visualized in TH) diameters [32, 33].

It must be noted that the use of the IVC-DI as primary outcome can be challenged. There is still ambiguity in the literature on which index should be applied to patients ventilated in PSV. In a post-hoc analysis comparing the IVC-CI and using the cut-off commonly adopted in spontaneously breathing patients, our whole cohort would have been identified as not being FR, which is clinically unlikely (patients in weaning and most commonly without noradrenaline support). Therefore, we think that choosing the IVC-DI was the most adequate approach. and Juhl-Olsen et al. demonstrated that IVC-CI cannot be used as an estimate of FR in both SC and TH views in patients in positive pressure ventilation [34].

We think our study has also the value on warning clinicians that IVC assessment in different projections and modalities is not interchangeable, and this assumes more importance in the evolving ICU landscape with shift towards reduced sedation and increased spontaneous breathing [35–37]. Until now, there was scarce evidence about the clinical utility of IVC assessment for FR in patients ventilated in PSV. However, it must be clear that other commonly utilized dynamic indexes as stroke volume variation and pulse pressure variation have shown poor prediction of FR in the transition from fully controlled mechanical ventilation to the PSV and spontaneous breathing modes [38].

We speculated that ABT technology might reproduce the standard M-mode IVC measurements, bringing the additional value in enhancing everyday clinical practice, with the option of faster and multiple measurements and reduced clinical workload; however, our results point in a different direction. One study reported that machine learning generated multiple models for the prediction of FR and these were comparable with the hemodynamic findings of passive leg raising. Among other parameters, the IVC CI was one of the variables supported by the model in addition to Doppler measurements such as velocity–time integral, S-wave, E/Ea ratio, and E-wave [39]. Despite deep learning algorithms enabled of IVC video classification with the aim to evaluate FR have demonstrated moderate performances by the trained algorithm (AUC 0.70) [23], the same group of authors demonstrated that algorithms used for implementation of ABT for IVC measurements are significantly influenced by the image definition, with worse results obtained with images of lower definition [40].

### Strengths and limitations

The main strength of our study is its factorial 2 × 2 design, which allowed for cross-validity of our measurements with a reference method. This study was conducted in a homogenous cohort intubated and assisted in PSV with their own respiratory trigger and on stable hemodynamic conditions, with roughly one third of patients on a single drug for hemodynamic support (norepinephrine, mostly low dose).

Our study has several limitations. First, the study did not allow entire blinding of the operator as the ABT calculation is obtained in real-time. However, we minimized the impact of this issue as the M-mode calculations were performed off-line. Second, we did not assess FR and therefore we cannot extrapolate a cut-off regarding this aspect, and our study remains an investigation limited to the comparison of results between different acquisition sites (SC or TH) and calculation modes (M-mode or ABT). Third, despite the operator taking measurements has advanced echocardiography experience, the absence of other operators limits the external validity of the study. Fourth, this study was performed with a single manufacturer ABT equipment. There might be differences in other vendors' tracing technology, finally resulting in different precision and accuracy. Our findings hold true for the currently available technology, and we might expect a dynamic development in advanced ABT. Fifth, this study evaluated a standard approach to measuring IVC-DI with M-mode and ABT. Because IVC demonstrates an anisotropic change, single-point and single-axis measurements might not reflect the true IVC size changes, and the use of 2-D assessment with IVC area may produce better results. Although sounding, the evaluation of IVC area change has been less studied and deserve further investigation. Sixth, we did not perform an assessment of respiratory efforts. All measurements were taken during a period of a quite breathing with a standardized tidal volume of 6–8 ml/kg of predicted body weight, but no formal assessment of respiratory effort (i.e. P0.1), esophageal pressure or diaphragmatic excursion were utilized. Seventh, though patients were on steady hemodynamic and neurological condition, we did not record the sedation scores and depth of breathing may influence results [27].

## Conclusions

In critically ill patients with assisted breathing in pressure support ventilation, we found that the measurements of the inferior vena cava distensibility index calculated with M-mode or automated border tracking software are not interchangeable, due to suboptimal precision. Moreover, the inferior vena cava distensibility index gathered from subcostal or transhepatic view produces not comparable results in regards to accuracy and precision.

## Supplementary Information

Below is the link to the electronic supplementary material.

Supplementary file1 (DOCX 18 KB)

## Data Availability

No datasets were generated or analysed during the current study.
