# Supplementary material for: Inferior vena cava distensibility during pressure support ventilation: a prospective study evaluating interchangeability of subcostal and trans‑hepatic views, with both M‑mode and automatic border tracing
Source: J Clin Monit Comput. 2024 May 31;38(5):981–90. doi: 10.1007/s10877-024-01177-8 (PMC11427491; doi:10.1007/s10877-024-01177-8)
Supplement: Supplementary file 1 — Supplementary file1 (DOCX 18 KB) [file 10877_2024_1177_MOESM1_ESM.docx]

**SUPPLEMENTARY MATERIAL**

1. Main diagnosis of admission and severity score (SOFA Score)

| **n** | **Diagnosis of admission** | **SOFA Score** |
| --- | --- | --- |
|  | AKI on CKD | **10** |
|  | Coma | **9** |
|  | GVHD | **14** |
|  | Traumatic brain injury | **10** |
|  | Myocardial infarction | **11** |
|  | Pancreatitis | **14** |
|  | ROSC | **4** |
|  | Subarachnoid hemorrhage | **9** |
|  | AKI | **15** |
|  | Septic Shock + Aortic Dissection | **11** |
|  | Caustic Ingestion | **16** |
|  | Hemorrhagic Shock | **14** |
|  | Subarachnoid hemorrhage | **6** |
|  | Encephalitis | **13** |
|  | ROSC | **16** |
|  | ROSC + ARDS | **15** |
|  | ARDS | **7** |
|  | MOF | **11** |
|  | ARDS | **18** |
|  | Stroke | **11** |
|  | Postoperative monitoring | **9** |
|  | ARDS | **11** |
|  | Traumatic brain injury | **10** |
